# Protocol for determining the regulation of lipid kinases and changes in phospholipids *in vitro*

**DOI:** 10.1016/j.xpro.2021.100926

**Published:** 2021-11-01

**Authors:** Cansu Karabiyik, David C. Rubinsztein

**Affiliations:** 1 Department of Medical Genetics, Cambridge Institute for Medical Research, University of Cambridge, Cambridge CB2 0XY, UK; 2 UK Dementia Research Institute, Cambridge, UK; 3 Mortimer B. Zuckerman Mind Brain and Behavior Institute, Columbia University, 3227 Broadway, Manhattan, NY 10027, USA

**Keywords:** Cell Biology, Metabolism, Signal Transduction, Protein Biochemistry, Protein expression and purification

## Abstract

The regulation of lipid kinases has remained elusive given the difficulties of assessing changes in lipid levels. Here, we describe the isolation of protein and lipid kinases to determine the regulation of lipid kinases *in vitro*. This can be followed by analysis of effects of regulators on lipid kinase-mediated changes in phospholipids without the use of radioactivity, with a specific focus on PI(5)P generation by the enzyme PIKfyve.

For complete details on the use and execution of this protocol, please refer to Karabiyik et al. (2021).

## Highlights

• Isolation of proteins to determine the regulation of lipid kinases *in vitro*
• Assessing changes in lipid kinase activity by visualization of lipid products
• Monitoring of lipid kinase activation by upstream effectors and the downstream effects
• Non-radioactive assay to detect changes in phospholipid levels

## Graphical abstract

**Figure:**
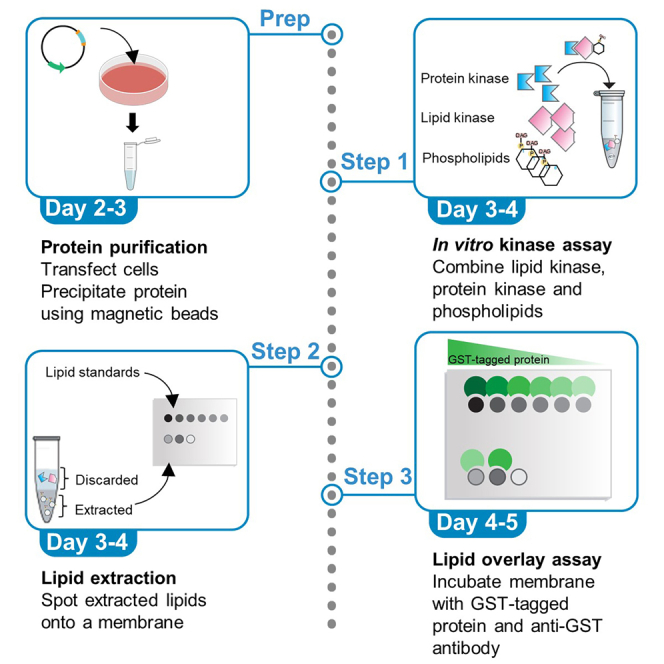

## Before you begin

This protocol describes the specific steps that allow assessment of the regulation of the lipid kinase activity of PIKfyve. This protocol can be used to study the regulation of various lipid kinases. Phosphatidylinositol (PI) lipids are abundant components of eukaryotic cell membranes. As a response to upstream signals, lipid kinases phosphorylate PI on the inositol head group and generate an array of lipid second messengers. The phosphorylated lipids regulate the activity of a variety of proteins, including protein kinases, ion channels and adaptor proteins (Di Paolo and De Camilli, 2006). To study the enzymatic properties of lipid kinases, these are often purified and assayed using radiolabeled lipids (Sbrissa et al., 2002). Here, we present a method to study the regulation of lipid kinases in a non-radioactive manner. In our recent study (Karabiyik et al., 2021), we examined the regulation of the lipid kinase PIKfyve by an upstream mediator, the autophagy regulator ULK1.

PIKfyve generates PI(3,5)P_2_ and PI(5)P lipids by phosphorylating the D-5 position in PI(3)P or PI (Shisheva et al., 1999), which play important roles in various cellular functions (Shisheva et al., 2015). We explored whether ULK1-mediated phosphorylation of PIKfyve would increase PI(5)P levels. First, we performed an *in vitro* kinase assay containing PIKfyve and ULK1 to allow phosphorylation of PIKfyve by ULK1 in the presence or absence of an ULK1 inhibitor or a PIKfyve inhibitor. Next, PI lipids were added to the reaction to allow phosphorylation of PIs by PIKfyve. The reaction was terminated by extracting phospholipids following an approach described by Shisheva et al. (2001). The lipid extracts were spotted on a nitrocellulose membrane for a protein lipid overlay assay. To visualize changes in PI(5)P levels in the various reactions without the use of radioactive material, we exploited the fact that PI(5)P is bound by the plant homeodomain of the ING2 protein as a rationale for visualization of PI(5)P lipids (Gozani et al., 2003). The membrane spotted with lipids was incubated with recombinant GST-tagged ING2 protein following an approach previously described by Dowler et al. (2002). PI(5)P present on the membrane should be bound by GST-ING2. By immunoblotting the membrane with an anti-GST antibody and secondary antibodies, changes in PI(5)P lipids in the various reactions were visualized, thus allowing us to infer how PIKfyve activity was regulated by ULK1. We have used this protocol to effectively study changes in PI(5)P levels by assessing GST-ING2 binding.

The same rationale can be applied to study the regulation of any lipid kinase whose lipid products are known, and which have suitable bio-probes. Before proceeding with this protocol, considerations should be made on downstream effectors, depending on the lipid kinase of interest (see below).

### Optimization of transfection

**Figure 1:**
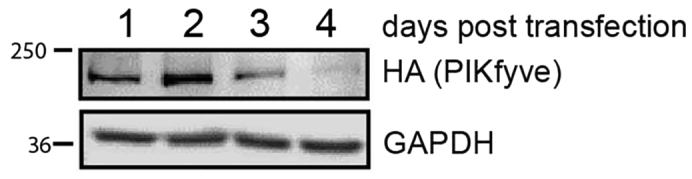
Optimization of transfection HEK 293T cells were transfected with HA-PIKfyve. Cells were lysed on day 1, 2, 3 or 4 post transfection. Immunoblots were blotted with anti-HA antibody. GAPDH was used as loading control.

To gain the highest yield of proteins, the use of HEK 293T cells is recommended. First, a control experiment should be performed to determine which day post transfection offers the highest protein yield. In this study, 4 wells of a 6 well plate were seeded with HEK 293T cells. The following day, all wells were transfected with HA-PIKfyve. We recommend transfecting one well with an empty backbone vector. Each day, the contents of one well were lysed with lysis buffer. After collection of all wells, the protein concentration in each lysate was determined with a BCA protein assay (as per manufacturer’s instructions). Lysates (20 ug protein/well) were resolved with SDS-PAGE. Our study found that the highest yield was achieved on day 2 post transfection of cells with a plasmid containing HA-PIKfyve (Figure 1).

### Identification of lipid binding partners

A protein lipid overlay assay will be performed to assess the effects of the regulation of a lipid kinase on its synthesis of given phospholipids. To this end, the product of the lipid kinase should be identified, and a binding protein selected for use in this protocol.

Many proteins contain a lipid binding domain, such as pleckstrin homology (PH) domain; the Fab1p, YOTB, Vac1p, EEA1 (FYVE) domain; the PhoX homology (PX) domain; the Epsin NH2-terminal homology (ENTH) domain; the glucosyltransferase, Rab-like GTPase activator and myotubularin (GRAM) domain; the cytosolic N-terminal polybasic domain of the mucolipin 1 channel (MLN1) (Di Paolo and De Camilli, 2006; Li et al., 2013). A list of phospholipids with their known interactors is shown in Table 1.

**Table 1:** Phosphoinositide-binding domains

Phospholipid	Lipid kinase	Lipid-protein binding	Domain
PI(3)P	Class III PI(3)kinase hVPS34	EEA1, Hrs, SARA, PIKfyve	FYVE
		SNX2, 3, 7, 13	PX
PI(3,5)P_2_	PIKfyve (PIP kinase Type III)	Myotubularin	GRAM
		Ent3p, Ent5p	A/ENTH
		TRPML1	MLN1
PI(3,4)P_2_	5-phosphatase SHIP2	AKT, TAPP1,2	PH
		P47phox	PX
PI(3,4,5)P_3_	Class I PI(3)kinase p110a	BTK, AKT, ARNO, GRP1	PH
		SHC	PTB
		CISK	PX
PI(4)P	PtdIns(4)kinase Type IIa, 5-phosphatase OCRL, polyphosphoinositide phosphatase	EpsinR	A/ENTH
		FAPP1/2, OSBP	PH
PI(4,5)P_2_	PIP kinase Type IIa, 3-phosphatase PTEN	AP180, CALM, epsin, HIP	A/ENTH
		Synaptotagmin	C2
		Ezrin, moesin, radixin, talin	FERM
		Syntenin	PDZ
		PLC, dynamin	PH
		Class II PI(3)K	PX
PI(5)P	PIKfyve, 3-phosphatase myotubularin-related protein (MTMR2)	ING2	PHD
		SNX13	PX

An overview of phospholipids, their known interactors, and the binding domains. PH, Pleckstrin homology; FYVE, Fab1p, YOTB, Vac1p, EEA1; PX, PhoX homology; ENTH, Epsin NH2-terminal homology; GRAM, glucosyltransferase, Rab-like GTPase activator and myotubularin; MLN1, cytosolic N-terminal polybasic domain of the mucolipin 1 channel.

Table 1 should be used to select the appropriate lipid binding protein for visualization of changes in phospholipids. e.g., The FYVE domain specifically binds PI(3)P lipids and would be an appropriate domain to visualize changes in PI(3)P lipids. The PH or PX domains bind a variety of lipids and would therefore confound findings if used in this protocol.

**Figure 2:**
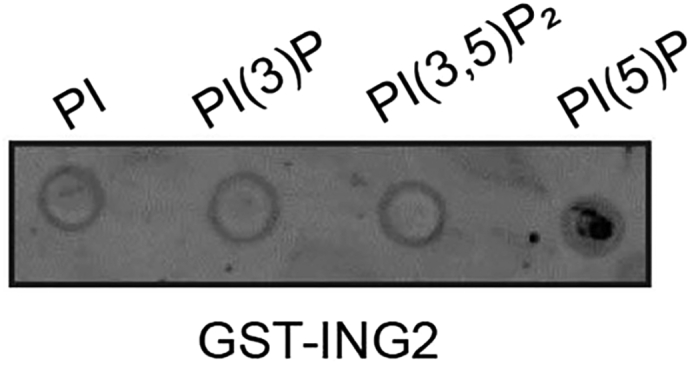
ING2 binds PI(5)P Exogenous PI, PI(3)P, PI(3,5)P_2_ or PI(5)P (100 μM) were spotted on a membrane. The membrane was incubated with recombinant GST-ING2 (10 nM). Anti-GST antibody was used to visualize GST-ING2 binding to the lipids on the membrane. Where necessary, consider comparing signals from GST-tagged lipid probe to GST alone.

In our study, we used binding to the ING2 protein to assess changes in PI(5)P levels. We tested whether GST-ING2 bound to PI, PI(3)P, PI(3,5)P_2_ or PI(5)P lipids and found that it specifically bound PI(5)P (Figure 2). We suggest performing a similar test prior to employment of the lipid binding protein in this assay.

### Perform a preliminary *in vitro* kinase assay

In our study, we assessed the ULK1-mediated effects on the lipid kinase activity of PIKfyve. Prior to this, we established that ULK1 phosphorylates PIKfyve by performing a malachite green-based *in vitro* kinase assay, determining the concentration and time required for ULK1 phosphorylation of PIKfyve. We suggest establishing these parameters for the protein kinase of interest prior to conducting the assay presented here.

## Key resources table

**Table:** 

REAGENT or RESOURCE	SOURCE	IDENTIFIER
**Antibodies**		

anti-GST antibody [3G10/1B3]	Abcam	#ab92 RRID:AB_307067
anti-HA.11 clone 16B12	Covance	#2729S RRID:AB_1031062
Goat anti-Rabbit IgG (H+L) Secondary Antibody, DyLight 680	ThermoFisher	#35568

**Chemicals, peptides, and recombinant proteins**		

SBI-0206965	Sigma Aldrich	#SML1540
YM206136	Merck	#524611
PI(5)PdiC16	Echelon	# P-5016
PIdiC16, PI(3)PdiC16, PI(3,5)P2diC16	Echelon	#P-0016 #P-3016 #P-3516
Recombinant Human ULK1	ThermoFisher	#PV6430
Recombinant Human-ING2	Creative Biomart	#5130H
ATP 100mM solution pH 7.5	Sigma-Aldrich	#GE27-2056-01
cOmplete Protease Inhibitor Cocktail	Sigma-Aldrich	#11697498001
Phosphatase Inhibitor Cocktail 2	Sigma-Aldrich	#P5726
Phosphatase Inhibitor Cocktail 3	Sigma-Aldrich	#P0044
InstantBlue Coomassie Protein Stain	Abcam	#ab119211

**Critical commercial assays**		

Universal In Vitro Assay Kit	R&D Systems	#EA004
Pierce HA magnetic beads	ThermoFisher	#88836
Pierce BCA Protein Assay Kit	ThermoFisher	#23225

**Experimental models: Cell lines**		

HEK 293T	ATCC	#CRL-3216; CVCL_0063

**Recombinant DNA**		

pCMV5-HA-PIKfyve WT	A. Shisheva	n/a

**Software and algorithms**		

Image Studio Lite	LI-COR	n/a

**Other**		

Hybond C-extra nitrocellulose membrane	GE Healthcare	#RPN1520N
Glass vials	Analytical-sales	#88120-CASE
Nonstick, RNase-free Microfuge Tubes, 1.5 mL	ThermoFischer	#AM12450

## Materials and equipment

**Table:** Lysis buffer

Reagent	Final concentration	Amount
NP-40 (10%)	0.5%	1 mL
Tris pH 7.5 (1 M)	50 mM	1 mL
NaCl (5 M)	150 mM	600 μL
EDTA (0.5 M)	0.5 mM	20 μL
Protease inhibitors 100×	1×	200 μL
Phosphatase inhibitors 100×	1×	200 μL
ddH_2_O	n/a	16.9 mL
**Total**	**n/a**	**20 mL**

**CRITICAL:** Keep buffer on ice. Buffer can be prepared and kept in −20°C. Phosphatase and protease inhibitors should be added immediately before use.

***Note:*** Additives such as 1mM DTT may be added to lysis buffer for optimal kinase stability.

**Table:** Wash buffer

Reagent	Final concentration	Amount
Tris pH 7.5 (1 M)	50 mM	1 mL
NaCl (5 M)	150 mM	600 μL
EDTA (0.5 M)	0.5 mM	20 μL
ddH_2_O	n/a	18.4 mL
**Total**	**n/a**	**20 mL**

***Note:*** Buffer can be stored in 4°C for up to a month.

**Table:** RIPA buffer

Reagent	Final concentration	Amount
Triton-X (10%)	1%	5 mL
Tris pH 8.0 (1 M)	50 mM	2.5 mL
NaCl (5 M)	150 mM	1.5 mL
EDTA (0.5 M)	0.5 mM	20 μL
sodium deoxycholate (10%)	1%	2.5 mL
SDS (sodium dodecyl sulfate) (20%)	0.1%	250 μL
ddH_2_O	n/a	38.5
**Total**	**n/a**	**50 mL**

**CRITICAL:** Sodium deoxycholate stock must be protected from light. RIPA buffer stock can be stored in aliquots at −20°C.

**Table:** Buffer A

Reagent	Final concentration	Amount
HEPES pH 7.4 (1 M)	50 mM	2.5 mL
NaCl (5 M)	150 mM	1.5 mL
EDTA (0.5 M)	1 mM	10 μL
ddH_2_O	n/a	46 mL
**Total**	**n/a**	**50 mL**

***Note:*** Buffer can be stored in 4°C for up to 2 months.

**Table:** Buffer B

Reagent	Final concentration	Amount
Tris-HCl pH 7.5 (1 M)	100 mM	5 mL
LiCl (5 M)	500 mM	5 mL
ddH_2_O	n/a	50 mL
**Total**	**n/a**	**50 mL**

LiCl ensures effective removal of non-specific chromatin interactions with the agarose beads.

***Note:*** Buffer can be stored in 4°C for up to 2 months.

**Table:** Buffer C

Reagent	Final concentration	Amount
Tris-HCl pH 7.5 (1 M)	10 mM	0.5 mL
NaCl (5 M)	100 mM	1 mL
EDTA (0.5 M)	1 mM	10 μL
ddH_2_O	n/a	48.5
**Total**	**n/a**	**50 mL**

***Note:*** Buffer can be stored in 4°C for up to 2 months.

**Table:** Assay buffer

Reagent	Final concentration	Amount
HEPES pH 7.4 (1 M)	25 mM	1.25 mL
NaCl (5 M)	120 mM	1.2 mL
MnCl_2_ (0.5 M)	2.5 mM	25 μL
MgCl_2_ (0.5 M)	2.5 mM	25 μL
β-glycerophosphate (1 M)	5 mM	25 mL
DTT (100 M)	1 mM	50 μL
ddH_2_O	n/a	47.4
**Total**	**n/a**	**50 mL**

β-glycerophosphate is added to inhibit potential immunoprecipitated phosphatases that may interfere with the assay.

***Note:*** Prepare fresh before use.

**Table:** Lipid buffer

Reagent	Final concentration	Amount
HEPES pH 7.4 (1 M)	20 mM	1 mL
EDTA (0.5 M)	1 mM	10 μL
ddH_2_O	n/a	49 mL
**Total**	**n/a**	**50 mL**

***Note:*** Buffer can be stored in 4°C for up to 2 months.

**Table:** TBS-T buffer

Reagent	Final concentration	Amount
TBS (10×)	1×	100 mL
Tween 20	0.1%	1 mL
ddH_2_O	n/a	900 mL
**Total**	**n/a**	**1 L**

**Table:** Blocking buffer

Reagent	Final concentration	Amount
TBS-T 0.1%	n/a	50 mL
BSA	2 mg/mL	100 mg
**Total**	**n/a**	**50 mL**

***Note:*** Prepare fresh prior to use.

## Step-by-step method details

### Precipitation of protein from mammalian cells

**Timing: 3 days (depends on optimal transfection day)**

1. Purify proteins (kinase and/or substrate) using HEK 293T cells. This may be omitted if commercial recombinant proteins are available.
  a. Seed HEK 293T cells in 140 mm cell culture dishes.
  b. Transfect cells with plasmid containing gene of interest with tag:
    i. One/two days later, wash cells with PBS three times and lyse in ice-cold lysis buffer.
    ii. Incubate lysates on ice for 30 min.
    iii. Centrifugate at 16,100g for 10 min at 4°C.
    iv. In the meantime, wash magnetic beads three times with wash buffer using a magnet.
    v. Incubate post-nuclear supernatant with magnetic beads at 4°C on a rotating wheel for 1–2 h (time depends on the manufacturer’s instructions).
    vi. Centrifugate briefly and remove supernatant. Wash the beads three times with wash buffer. Mix by inverting tubes.
      ***Note:*** If working with large quantities, proteins may be eluted using a peptide that competes with the bead-protein binding.
      Protein purity should be assessed using Coomassie or Silver stained gels, which can also be used to estimate protein concentrations by adding one or more lanes with defined amounts of a protein of known concentration as described below in c.
  c. Recommended. Determine protein concentration of the bead-bound protein:
    i. Prepare a stock solution of BSA (10 mg/mL).
    ii. Prepare 7 dilutions of BSA.
    iii. Add 2× Laemmli buffer with β-mercaptoethanol to 10 μL of each BSA solution and 10 μL of bead-bound protein. After boiling samples at 100°C for 5 min, run samples on SDS–PAGE at 100 V for 2 h.
    iv. Incubate the gel with Coomassie Blue stain to determine the relative protein amount bound by the beads and ensure that additional bands are not present.
    v. Wash bead-bound protein with buffers as described below.
      **CRITICAL:** If large amounts of protein have been precipitated, aliquot and store at −80°C to avoid freeze and thaw. We suggest using magnetic beads. Agarose resin beads may be used if protein is eluted from the beads. However, we do not suggest using agarose resin-tagged protein for further steps, as the agarose resin cannot be washed off the membrane used in future steps.
      ***Note:*** While in this study, we used a precipitated substrate (HA-PIKfyve) and a recombinant commercial kinase (ULK1) without experiencing technical issues, this step may be skipped if both proteins are available as commercial products. Alternatively, both proteins may be purified using HEK 293T cells.
      ***Note:*** Since the relative ratio of substrate to kinase may affect the outcome of the kinase activity, we recommend determining the concentration of precipitated substrate and performing a kinase assay with varying concentrations of substrate to determine the optimal conditions.

### *In vitro* lipid kinase assay

**Timing: 1 h**

In this step an *in vitro* kinase assay was performed assessing whether the ULK1-mediated phosphorylation of PIKfyve leads to an increase in the lipid kinase activity of PIKfyve. Prior to this, we performed a malachite green-based *in vitro* kinase assay and found that ULK1 phosphorylates PIKfyve. Here, we assess whether ULK1 phosphorylation of PIKfyve leads to an increase in the lipid kinase activity of PIKfyve.

2. Prepare phosphoinositides for the kinase assay:
  a. Prepare a 1:1 solution of methanol and chloroform.
  b. Add to glass vial containing PI or PI(5)P lipids for a 1 mM stock. Glass vials will minimize sticking that can occur to plastic tubes.
  c. Add PI solution to empty tubes (n tubes depends on the number of reactions) for a final concentration of 100 μM.
  d. Dry off the chloroform using an argon gas flux until all liquid has evaporated and a film is visible at the bottom of the tube.
  e. Reconstitute lipid in lipid buffer.
  f. Sonicate twice for 30 s on ice.

3. Prepare kinases:
  a. Wash the precipitated proteins three times with RIPA buffer, twice with buffer A, three times with buffer B, twice with buffer C and twice with assay buffer.
  b. Incubate lipid kinase, protein kinase (10 nM) and ATP (50 μM) in assay buffer (total volume 50 μL) for 15 min at 37°C in a non-adherent tube.
  c. Next, add the reaction mix to tube containing PIs (100 μM). Incubate for additional 15 min at 37°C.
    ***Note:*** The time points are subject to change depending on the kinase.
  d. Set up the reactions in Table 2:

4. Stop the reaction by adding 200 μL 1N HCl. The lipids should be used directly in the next step as phospholipids may be unstable when frozen. If required, we recommend storing lipids in an organic solvent such as chloroform in a tightly sealed glass vial and storing at −20°C.

**CRITICAL:** The purified kinases should be as pure as possible. The wash steps will ensure dissociation of interactors. To limit confounding variables, SDS-PAGE may be performed for the precipitated kinases, assessing whether additional bands are visible after a Coomassie Blue stain as mentioned earlier.

**CRITICAL:** If inhibitors of the protein kinase and/or the lipid kinase are available, these should be included in the assay as additional controls. Inhibitors and kinases should be incubated prior to initiation of the kinase assay.

***Note:*** If the phosphorylation sites are known, we suggest using phospho-mutant and phospho-mimic forms of the lipid kinase to further validate the effect of the protein kinase on the lipid kinase.

***Note:*** The SDS and sodium deoxycholate in the RIPA buffer may cause partial denaturation of some proteins. While this was not observed in our study, compromised activity of certain enzymes caused by these detergents in RIPA buffer may necessitate the use of non-ionic detergents instead. The use of negative and positive controls would help ensure that a lack in activity is not caused by buffer compositions.

***Note:*** EDTA may interfere with the activity of some kinases. If kinase activity is too low, adjust buffers accordingly.

***Note:*** Some kinases may depend on accessory proteins for activation of their kinase function. Orthogonal tools should be employed to assess the requirement for additional proteins.

**Table 2:** Recommended reactions

	Lipid kinase	Protein kinase	PI	ATP
Tube 1	+	-	+	+
Tube 2	+	+	+	+
Tube 3	-	+	+	+
Tube 4	+	+	-	+

An overview of reactions that contain lipid kinases, protein kinases, phosphoinositides (PI) and ATP with appropriate controls.

### Lipid extraction

**Timing: 1 h, 20 min**

If the lipid kinase has become activated in the *in vitro* kinase assay, this should lead to a generation of phospholipids.

5. Extract lipids from the kinase reaction:
  a. Add 160 μL 1:1 chloroform:methanol to the tube containing the reaction to create a biphasic system.
  b. Vortex and centrifuge tube at 10,000g for 2 min.
  c. Remove the upper methanol layer containing the kinases.
  d. Wash the lower chloroform layer twice with 100 μL of 1:1 of methanol:1N HCl.
  e. Use an argon gas flux to remove excess volume.
  f. Apply the resulting lipid solution left in the tube to a nitrocellulose membrane.
6. According to the lipid of interest, prepare the following steps:
  a. Dilute lipids in a 2:1:0.8 solution of methanol:chloroform:water in 5–8 serial dilutions starting with 100 μM.
  b. Spot 5 μL of each dilution on the same nitrocellulose membrane as the lipids extracted from the kinase reactions.
7. Air-dry the membrane at room temperature for 1 h.

***Note:*** To prevent the membrane from curling upon spotting with lipids, tape it firmly to a flat surface. Once dry, use a clean razor blade to cut within the edges of the tape to use it for the next steps.

### Protein lipid overlay assay

**Timing: 1 day**

The objective of this part of the protocol is to assess changes in lipid synthesis as a result of the lipid kinase activation by the protein kinase of interest. This step requires knowing whether the lipid of interest is bound by any given protein (see above). In our study, we used GST-tagged ING2, as this protein is known to bind PI(5)P.

8. Incubate the membrane containing lipid spots in blocking buffer for 1 h at room temperature.
9. Incubate the membrane overnight at 4°C with 10 nM GST-ING2 protein in blocking buffer in 4°C.
  a. The next day, wash the membrane 10 × 5 min with TBS-T.
10. Incubate the membrane with a 1:2000 dilution of anti-GST monoclonal antibody in blocking buffer for 1 h at room temperature.
  a. Wash the membrane 10 × 5 min with TBS-T.
11. Incubate the membrane with secondary antibody for 1 h to visualize the GST-tagged protein bound to your lipid of interest. We used of DyLight Fluors-conjugated secondary antibody in TBS-T at 1:5000 for 1 h.
  a. Wash the membrane 10 × 5 min with TBS-T and 1 × 5min with TBS.
12. Visualize with direct infrared fluorescence detection using LICOR-Odyssey apparatus/Bio-Rad ChemiDoc.

***Note:*** If the products of the lipid kinase in question have yet to be characterized, this tool allows detection of generated lipids by performing serial dilutions of other phospholipids and repeating the above assay with tools that enable their identification and quantification - e.g., Table 1.

## Expected outcomes

**Figure 3:**
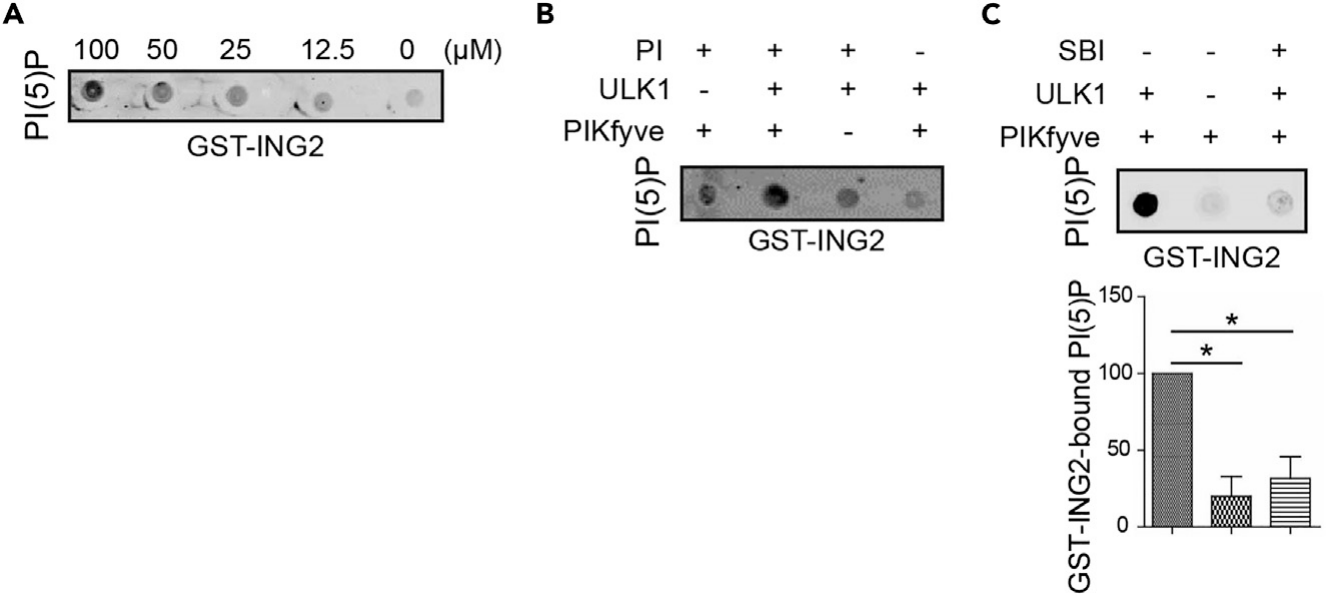
ULK1 causes a PIKfyve-mediated increase in PI(5)P lipids (A) Five serial dilutions of PI(5)P (100-0 μM) were spotted on a nitrocellulose membrane and incubated with GST-ING2 recombinant protein. PI(5)P-bound GST-ING2 was visualized by blotting membranes with anti-GST antibody. (B) HA-PIKfyve precipitated from HEK 293T using magnetic HA beads were incubated with PI (100 μM) and ATP (50 μM) ± recombinant ULK1 (10 nM; 15 min, 37°C). Lipids were extracted and spotted on a nitrocellulose membrane, which was treated as in (A). PI or HA-PIKfyve were not added to controls reactions as indicated. (C) HA-PIKfyve precipitated from HEK 293T using magnetic HA beads were incubated with PI (100 μM) and ATP (50 μM) ± recombinant ULK1 (10 nM; 15 min, 37°C) or following pre-treatment of ULK1 recombinant protein with the ULK1 inhibitor SBI-0206965 (SBI; 5 μM, 30 min). Lipids were extracted and spotted on a nitrocellulose membrane, which was treated as in (A). DMSO was used as vehicle control. Data show the relative levels of GST-ING2-bound PI(5)P represented as mean ± SEM (n = 3, ∗P < 0.05; two-tailed, paired student’s t-test).

If the protein kinase of interest leads to an activation of the lipid kinase activity, an increase in the generation of the relevant lipid should be observed. In our study, we found that the ULK1-mediated phosphorylation of PIKfyve led to an increased generation of PI(5)P lipids bound by GST-ING2. This was prevented with ULK1 inhibition (Figure 3).

While we did not assess the concentration of lipids synthesized by PIKfyve, this assay offers the option of quantitative measurement. This requires first determining the concentration of the precipitated lipid kinase. A concentration curve can be created by a densitometric quantification of the PI(5)P standards. Subsequently, using varying concentrations of lipid kinase in multiple reactions will allow one to determine kinase activity.

## Quantification and statistical analysis

Densitometric quantification of PI(5)P-containing spots can be performed for a quantitative analysis using FIJI/ImageJ or Image Studio Lite software. The relative amounts of GST-protein bound lipid in each reaction can be normalized to the reaction containing no protein kinase.

## Limitations

The success of this assay is determined by whether the conditions for protein kinase-mediated phosphorylation of the lipid kinase is established in preliminary assessments.

## Troubleshooting

### Problem 1

No protein is precipitated.

### Potential solution

The transfection efficiency may be too low and the transfection should therefore be optimized as described above.

The protein may not be correctly expressing the affinity tag. The plasmid should be sequenced to ensure that the sequence is in frame and in case of a C-terminal tag, that there are no stop codons in the sequence.

The lysis may have been inefficient. A number of reasons can affect the efficiency of lysis buffer. NP-40 in a 10% solution should not be kept for longer than a month. Ideally, fresh lysis buffer should be prepared each time.

If the protein-bead binding is too weak, the protein may be washed off the beads. The salt concentration may be adapted, so it is less stringent. Alternatively, a different affinity tag may be introduced by subcloning to ensure optimal binding.

### Problem 2

Lipid synthesis is not observed.

### Potential solution

Dissolve lipids prior to use. Do not keep aliquots of lipids that have been diluted in the 2:1:08 solution of methanol:chloroform:water in −20°C for longer than 2 weeks, as this may affect the assay.

If the lipid kinase used in the assay is bead-bound, it is possible that the beads may cause steric hindrance and prevent activation of the kinase. Elution of protein may resolve this issue (see above).

To ensure that the lack of lipid synthesis is not due to a technical issue, use a positive control. If an activator for the lipid kinase is available, it may be used to establish that the assay works.

### Problem 3

Low kinase activity.

### Potential solution

A time course should be performed to establish whether an increased reaction time is required for activation of the lipid kinase.

The effects of additives in the lysis buffer, such as DTT, may be tested to ensure optimal kinase activity as described above.

### Problem 4

High background.

### Potential solution

Membrane washes with TBS-T should be increased to 10 × 10 min. The final wash may be extended overnight to limit background.

## Resource availability

### Lead contact

Further information and requests for resources and reagents should be directed to and will be fulfilled by the lead contact, David C. Rubinsztein (dcr1000@cam.ac.uk).

### Materials availability

Materials generated in this study are available upon request.

## Data Availability

This study did not generate new datasets or code.
